# Ischemia Injury: A New Method Accelerates Bone Healing in a Rat Tibia Fracture Model

**DOI:** 10.1155/2019/6592464

**Published:** 2019-04-16

**Authors:** Yang Liu, Dong Wang, Xueqiang Wu, Junlin Zhou

**Affiliations:** Department of Orthopedics, Beijing Chaoyang Hospital, Capital Medical University, Beijing 100020, China

## Abstract

To find a simple and noninvasive method to promote fracture healing, we are trying to explore whether repetitive brief ischemia would promote bone healing. 88 rats divided into 6 groups were used to make right tibia closed fracture caused by the heavy weight collision method. Healthy side groups received homemade tourniquet placed on left and affected side group placed on right thigh 10 min inflated/10 min deflated 3 times every 24 hours or 48 hours after tibia fractured. Rats in control groups received homemade tourniquet uninflated placed on right thigh 1 hour every 24 hours or 48 hours. X-rays were checked at 1, 2, and 4 weeks. Micro-CT inspected the bone healing at 2 and 4 weeks. Serum cytokines, such as bone morphogenetic protein-2 (BMP-2), vascular endothelial growth factor (VEGF), diethanolamine enzyme activity unit of alkaline phosphatase (ALP) and transforming growth factor-*β*1 (TGF-*β*1), interleukin 10 (IL-10) and interleukin 6 (IL-6), were checked at 1, 2, and 4 weeks. Local histology was evaluated at 2 weeks. HE dye and BMP-2, VEGF, TGF-*β*, and ALP immunohistochemical stains were made. Callus areas of posterior-anterior and lateral views were calculated and repetitive brief ischemia increased the callus areas ratio at 1 and 2 weeks. Besides, from micro-CT results, repetitive brief ischemia increased the bone volume (BV) at 2 and 4 weeks and also increased the total bone tissue volume (TV) at 2 weeks and BV/TV at 4 weeks. The serum cytokines, such as BMP-2, VEGF, diethanolamine enzyme activity unit of ALP and TGF-*β*1, have increased by repetitive brief ischemia at 1, 2 weeks. It is opposite of affected side group that the level of serum IL-10 increased and IL-6 decreased in healthy side group at 1, 2 weeks. Repetitive brief ischemia increased the callus area at 2 weeks and boosted the synthesis of BMP-2, VEGF, TGF-*β*, and ALP in the fracture region at 2 weeks from tissue stains. Repetitive brief ischemia promotes bone healing no matter on the affected side or the healthy side limb.

## 1. Background

As accretion of osteoporosis population and traffic accidents rate increases, fracture becomes a common disease in society [1–3]. It is always a research hotspot to shorten bone healing time, relieve social burden, and restore labor ability of patients quickly [4–6]. However, few methods are inexpensive and widely available, though lots of ways including drugs and nondrugs are applied to promote bone healing. We are trying to explore whether repetitive brief ischemia would promote bone healing. Generally, tissue damage and cells death happen after ischemia, but repetitive temporary ischemia can relieve ischemia-reperfusion injury and enhance hypoxia tolerance of cells [7–10]. This study is to explore whether the bone healing accelerated if the healthy side limb received repetitive brief ischemia.

## 2. Materials and Methods

### 2.1. Animal

Female adult SD rats (Charles River Laboratories, China), 8 weeks old and weighing approximate 210g, were kept in separate cage with a controlled condition of 12 h light/12 h dark at 23.6°C, humidified at 35% and fed a sterilized chow diet with free water intake. All procedures were complied with the ARRIVE guidelines and carried out in accordance with the National Institutes of Health Guide for the Care and Use of Laboratory Animals. In addition, our study was approved by Capital Medical University Committee on the use of animals in research and education. At the end of the experiments, rats were euthanized by using excessive dose of pentobarbital sodium.

### 2.2. Surgical Procedures

At the beginning of the experiment, all animals underwent surgery to produce shaft closed fractures on right tibia. General anesthesia was obtained by intraperitoneal anesthesia with pentobarbital sodium (40 mg/kg body weight). The right hind leg of rat was shaved and disinfected by iodophor 3 times after successful anesthesia. Right shank was draped by sterile gauze and surgery was performed on small animal operating table under an aseptic condition.

**Figure 1:**
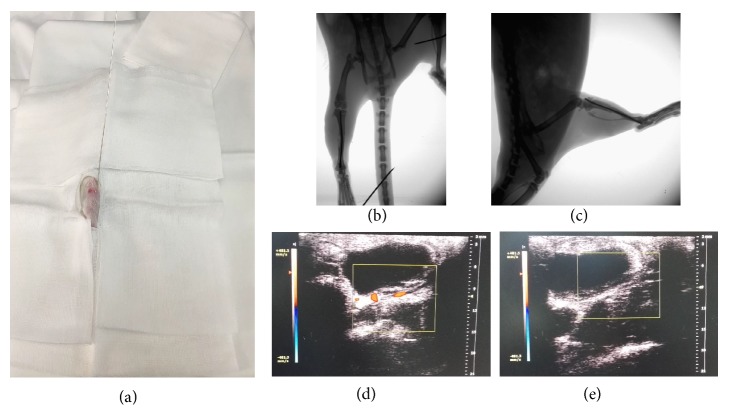
These images show the making process of fracture model and repetitive brief ischemia. (a) A sterilized 0.8 mm steel K-wire (Zimmer, USA) was used to enter the medullary cavity through the cortical bone by touching the leading edge of the tibial plateau when the keen was bent 90°. The wire stabbed directly the skin and was driven into medullary canal up to the distal of tibia. (b)-(c) X-ray examinations were performed to document the fracture reduction and the position of the implant ((b) anterior-posterior view; (c) lateral view). (d)-(e) Color doppler ultrasound (Vevo2100 imaging system, Canada) confirmed tourniquet pressure selected to block the blood ((d) tourniquet inflated; (e) tourniquet deflated).

The closed tibia fracture model was described in previous articles [7]. A sterilized 0.8 mm steel K-wire (Zimmer, USA) was used to enter the medullary cavity through the cortical bone by touching the leading edge of the tibial plateau when the keen was bent 90° (Figure 1(a)). The wire stabbed directly the skin and was driven into medullary canal up to the distal of tibia. A weight (500 g) was dropped at a distance of 35 cm upon the middle of tibia to make closed fracture model. The fracture was fixed with intramedullary 0.8-mm K-wire (Zimmer, USA) after closed reduction. The protruding part of wire was cut flush with the cortical surface. Then, X-ray examinations were performed to document the fracture reduction and the position of the implant (Figures 1(b) and 1(c)). Animals excluded due to comminuted fractures.

### 2.3. Experiment Design

Rats of the control group received homemade tourniquet uninflated placed on right thigh 1 hour every 24 hours (C24 group) or 48 hours (C48 group) after tibia fractured and healthy side groups received homemade tourniquet placed on left thigh 10 min inflated/10 min deflated 3 times every 24 hours (H24 group) or 48 hours (H48 group) after tibia fractured. Rats of the affected side groups were placed on right thigh every 24 hours (A24 group) or every 48 hours (A48 group). The pressure of the tourniquet was selected to block the blood flow and confirmed by color doppler ultrasound (Vevo2100 imaging system, Canada) (Figures 1(d) and 1(e)).

Stata12.0 was used to calculate the sample size and the data of previous preliminary experiments were adopted. The differences of callus area of posterior-anterior between C48 group and H48 group were the least among groups at two weeks and the differences of BV/TV between C48 group and H48 group were the least among groups at four weeks. Therefore, the above data was used for sample size calculation. Two-sample comparison of means was used and power of test was set at 0.8. Some rats were sacrificed at 2 weeks because of histological evaluation, so 88 rats were used at least.

### 2.4. Radiological Evaluation

The animals received X-ray examination at the end of experimental periods (1, 2, and 4 weeks, respectively). Rats were narcotized by intraperitoneal anesthesia with pentobarbital sodium (40 mg/kg body weight). Image station system (Bruker in vivo FX-PRO, China) was used to evaluate fracture healing by the illumination source adjusted to X-ray. Exposure time was set at 1min and posterior-anterior and lateral views were taken to evaluate the formation of callus. Adobe photoshop CS3 was used to pick region of interest (ROI) which was 5mm above and below fracture line; in addition, image-pro plus 6.0 analyzed the grayscale value of bone and callus and modified the color. Green represented callus and red was bone. Then, the ratio of area of callus to the bone tissue was calculated via image-pro plus 6.0.

Micro-CT (Bruker Skyscan 1176, Belgium) inspected the bone healing at 2 and 4 weeks. Rats were euthanized and lower limb was harvest. The K-wire was taken out before micro-CT examination. Source voltage was set 65kV and current was 381uA; besides, image pixel size was 18um and filter used Al 1mm. Rotation step was at 0.5° and 180° rotation was used. NRecon 1.6.10.2 (Bruker, Belgium) made two-dimensional reconstruction and region of interest (ROI) was 200 axial slices above and below the fracture line. Sequential images were obtained at pixel sizes of 18*μ*m and slice distances of 18um. Result image width and height were 2000 pixels. Mimics10.01 used three-dimensional reconstruction. Hounsfield units thresholding of bone volume (BV) was 226 to the max and total volume (TV) was -150 to the max. Magics9.9 calculated the BV and TV.

### 2.5. Cytokines Test

Bone morphogenetic protein-2 (BMP-2), vascular endothelial growth factor (VEGF), interleukin 6 (IL-6), diethanolamine enzyme activity unit of alkaline phosphatase (ALP), transforming growth factor-*β*1 (TGF-*β*1), and interleukin 10 (IL-10) in serum were checked at 1, 2, and 4 weeks. Rat BMP-2 ELISA kit, VEGF ELISA kit, IL-6 ELISA kit, TGF-*β*1 ELISA, IL-10 ELISA kit (Cusabio, China), and ALP test kit (Biyuntian, China) were used. Cytokines concentration and diethanolamine enzyme activity unit were obtained according to the user's instructions.

### 2.6. Histological Evaluation

Rats were euthanized by intraperitoneal excess pentobarbital and the K-wire was taken out at 2 weeks. Lower limb was harvest and bone tissue was decalcified by 5%EDTA, embedded in paraffin, sectioned at a thickness of 2 *μ*m along the longitudinal axis, and stored at 80°C until staining. HE dye as well as BMP-2, VEGF, TGF-*β*, and ALP immunohistochemical stain was made. First antibodies were rabbit anti-rat (BMP-2 1:200, VEGF 1:200, TGF-*β* 1:200 and ALP 1:200) (Biyuntian, China); in addition, second antibodies were goat anti-rabbit (1:50) (Biyuntian, China).

### 2.7. Statistical Analysis

Stata12.0 was used to analyze the data. Measurement data were reported as means ± deviation and enumeration data were reported as percentage. One-way ANOVA was used to compare the differences among the groups. Least significant difference (LSD) tests were used for multiple comparisons if variance is homogeneity, or Dunnett T3 test was used. P value <0.05 was accepted as statistically significant.

## 3. Results

### 3.1. X-Ray Evaluation

**Figure 2:**
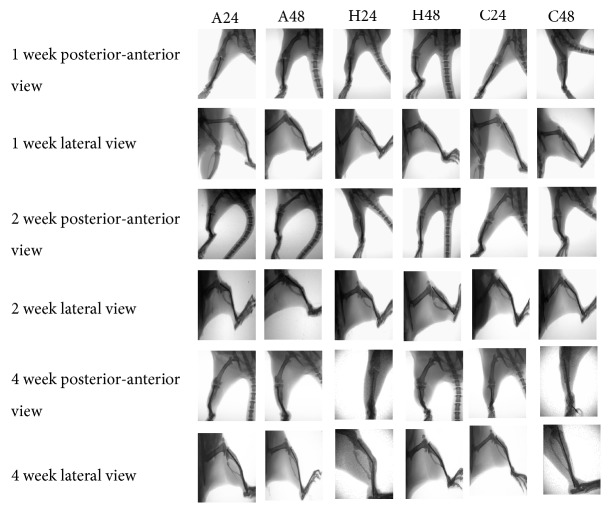
X-rays of rats in each group. Left side displayed the name of row and the top was the name of column. Affected limb received repetitive brief ischemia every 24 hours and every 48 hours (A24, A48), healthy limb received repetitive brief ischemia every 24 hours and every 48 hours (H24, H48), control group received homemade tourniquet uninflated every 24 hours and every 48 hours (C24, C48) from the left to the right and up to down in a chronological order. It can be seen from each image that repeated brief ischemia can significantly increase callus formation.

**Figure 3:**
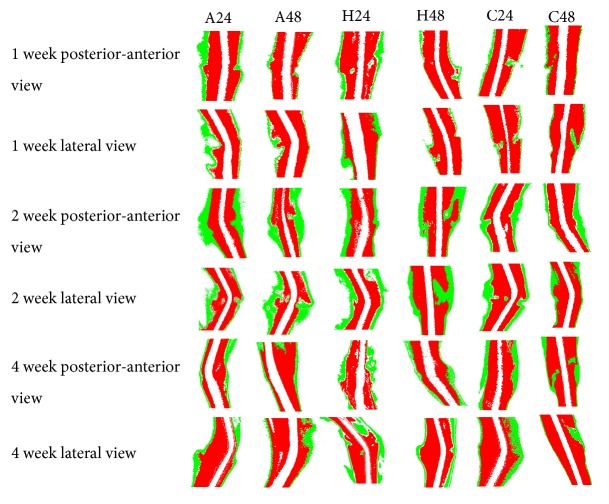
ROI bone tissue image. Region of interest (ROI) was 5mm above and below fracture line; in addition, image-pro plus 6.0 analyzed the grayscale value of bone and callus and modified the color. Green represented callus and red was bone. It can be seen from each image that repeated brief ischemia can significantly increase callus formation.

**Figure 4:**
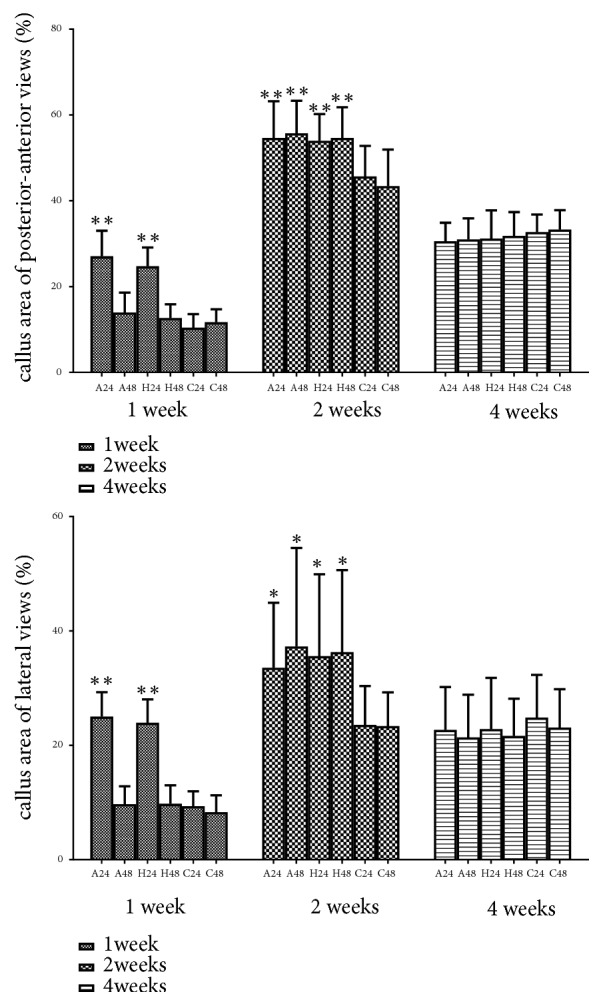
The callus area ratio in ROI of each group on X-rays. ROI was 5mm above and below fracture line. *∗*p<0.05, *∗∗*p<0.01.

Callus area of posterior-anterior and lateral views was calculated via image-pro plus 6.0 (Figures 2 and 3). Repetitive brief ischemia increased the callus areas ratio at 1 and 2 weeks. There was no statistical difference between each group (p>0.05) (Figure 4) (Table 1).

### 3.2. Micro-CT Evaluation

**Figure 5:**
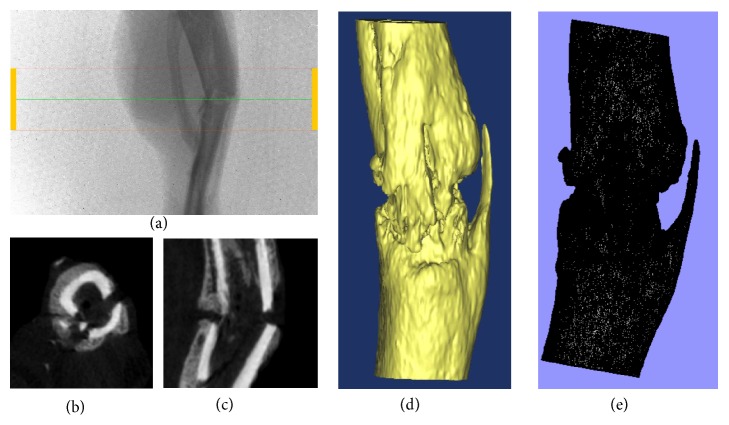
The process of micro-CT. (a)-(c) NRecon 1.6.10.2 (Bruker, Belgium) made two-dimensional reconstruction and region of interest (ROI) was 200 axial slices above and below the fracture line ((a) the ROI region; (b) horizontal plane; (c) sagittal plane); (d) three-dimensional reconstruction made by Mimics10.01; (e) the image in Magics9.9.

**Figure 6:**
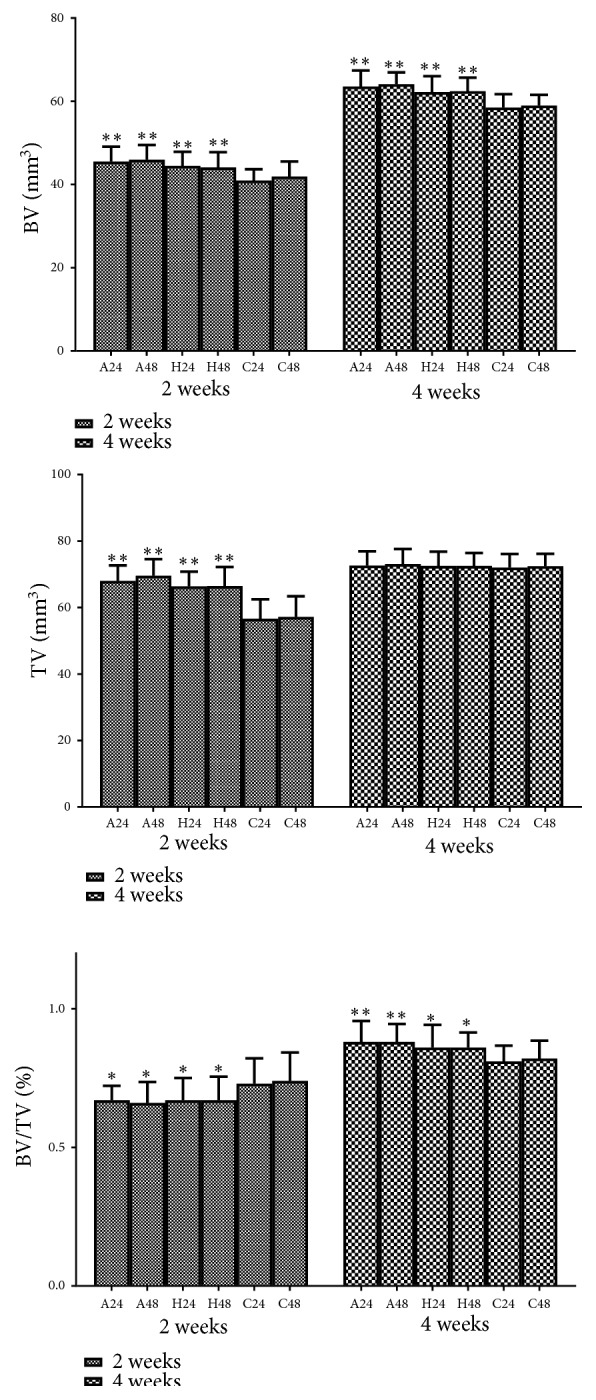
The results of micro-CT of each group. *∗*p<0.05, *∗∗*p<0.01.

BV, TV, and BV/TV were measured by Mimics10.01 and Magics9.9 (Figure 5). Repetitive brief ischemia increased the BV at 2 and 4 weeks. Besides, repetitive brief ischemia also increased the TV at 2 weeks and BV/TV at 4 weeks. There was no statistical difference between each group on TV at 4 weeks (p>0.05) (Figure 6) (Table 1).

### 3.3. Cytokines Results

**Figure 7:**
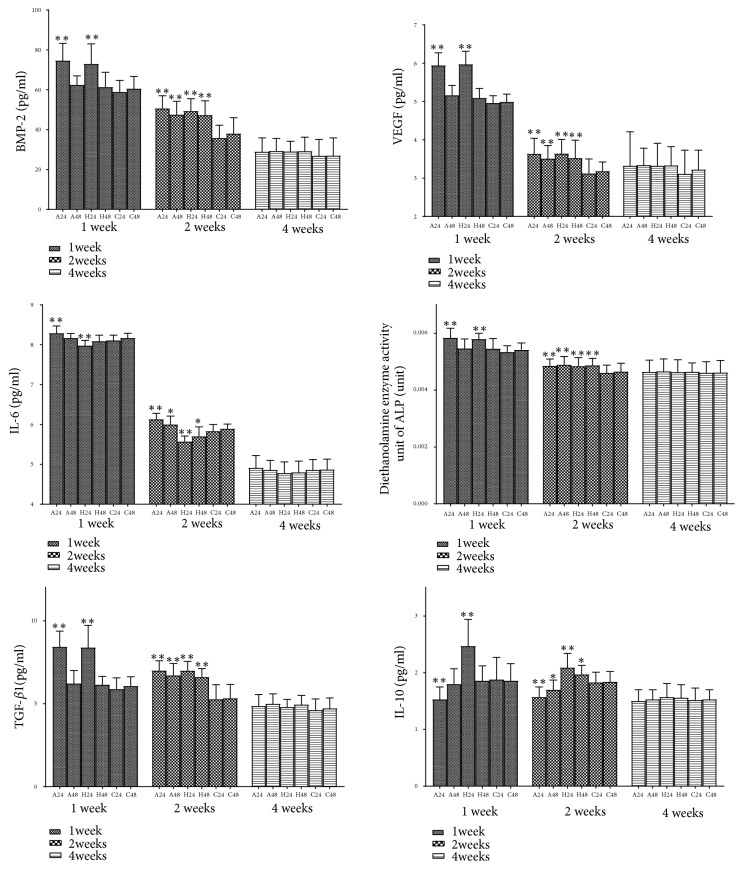
The cytokines of each group at 1, 2, and 4 weeks. *∗*p<0.05, *∗∗*p<0.01.

BMP-2, VEGF, IL-6, diethanolamine enzyme activity unit of ALP, TGF-*β*1, and IL-10 were detected by ELISA kit. The serum cytokines, such as bone morphogenetic protein-2 (BMP-2), vascular endothelial growth factor (VEGF), diethanolamine enzyme activity unit of alkaline phosphatase (ALP), and transforming growth factor-*β*1 (TGF-*β*1), increased by repetitive brief ischemia at 1,2 weeks. There was no statistical difference between each group at 4 weeks (p>0.05). Healthy side ischemia increased the level of serum interleukin 10 (IL-10) and decreased the level of interleukin 6 (IL-6), but affected side ischemia was the opposite at 1,2 weeks. There was no statistical difference between each group at 4 weeks (p>0.05) (Figure 7) (Table 1).

### 3.4. Histological Evaluation

**Figure 8:**
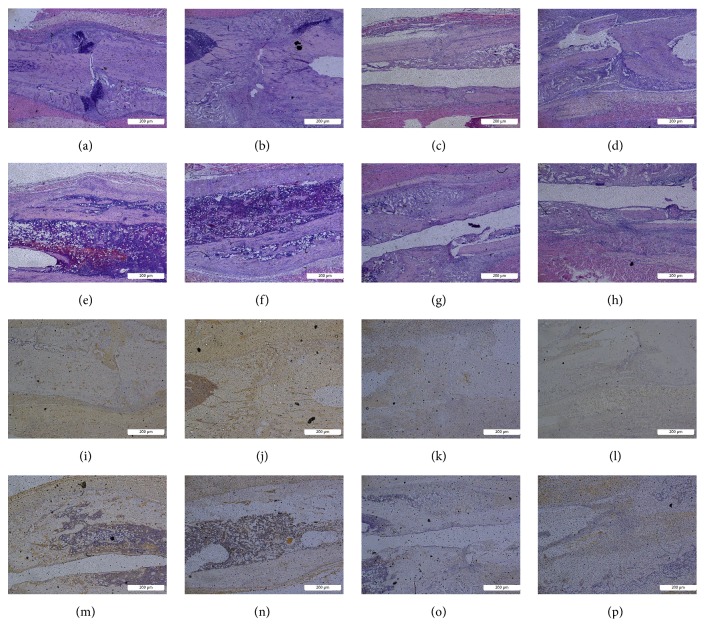
The HE dye and BMP-2, VEGF, TGF-*β*, and ALP immunohistochemical stain results. (a)-(h) HE stains and (i)-(n) immunohistochemical stains. (a)-(b) HE staining of fracture in early stage of rats that received repetitive brief ischemia. Visible callus formation can be seen in the pictures. (c)-(d) HE staining of fracture in early stage of control group rats. The amount of callus is significantly less than that of rats that received repetitive brief ischemia. (e)-(f) HE staining 4 weeks after fracture of rats that received repetitive brief ischemia. It can be seen that the callus has obvious ossification. (g)-(h) HE staining 4 weeks after fracture of control groups rats. The images showed that the degree of ossification in callus was significantly less that of rats that received repetitive brief ischemia. (i)-(j) Immunohistochemical stain of fracture in early stage of rats that received repetitive brief ischemia. A large number of cytokines synthetized can be seen at the fracture site ((i) BMP-2; (j) VEGF). (k)-(l) Immunohistochemical stain of fracture in early stage of control group rats. The synthesis of cytokines is less than that of rats that received repetitive brief ischemia ((k) BMP-2; (l) VEGF). (m)-(n) Immunohistochemical stain 4 weeks after fracture of rats that received repetitive brief ischemia. It can be seen that a large number of ossifications at the callus and the cytokines synthetized have been reduced ((m) TGF-*β*; (n) ALP). (o)-(p) Immunohistochemical stain 4 weeks after fracture of control group rats. Less ossification at the callus compared to rats that received repetitive brief ischemia and cytokines synthesized also less ((m) TGF-*β*; (n) ALP).

HE dye as well as BMP-2, VEGF, TGF-*β*, and ALP immunohistochemical stain was made. Repetitive brief ischemia increased the callus area at 2 weeks and boosted the synthesis of BMP-2, VEGF, TGF-*β*, and ALP at the fracture position at 2 weeks (Figure 8).

## 4. Discussion

Fracture is disrupting sclerotin composed of bone matrix and cells [11, 12]. Bone matrix and osteocyte originate to osteoblasts. When fracture happens, a variety of cells turn into osteoblasts in order to repair bone tissue [11, 13–15].

Nonunion sometimes occur in patients with lower tibia fracture due to the tissue ischemia. It is a heavy burden for the patient, family, and society due to the long healing time. How to accelerate bone healing has become a hot spot and our idea was from ischemic preconditioning and posttreatment [4–7].

Ischemia-reperfusion is a pathophysiology process after tissue ischemia and hypoxia induced vascular lesions and inflammatory reactions, but also has benefits [16–21]. Repetitive brief ischemia can reduce damage caused by promoting cytokines produced and increasing tissue tolerance to hypoxia [7, 22].

In previous animal experiments, rat right tibia fracture model was made by the heavy weight collision method [7]. Rats received repetitive brief ischemia on the right lower limb and bone healing shortened [7]. Then, repetitive brief ischemia was applied to patients with bone fracture and bone healing was also quickened. However, the sample size was small due to poor patient compliance. The reason is we thought it was difficult to cooperate with patients to let repetitive brief ischemia on the affected limb as they suffered pain. Therefore, we repeated the prior animal experiment and tested the serum cytokines at 1, 2, and 4 weeks. It was unexpected to find that bone morphogenetic protein (BMP), transforming growth factor-*β*1 (TGF-*β*1), and alkaline phosphatase (ALP) increased in animals receiving repetitive brief ischemia compared to rats without ischemia [7]. This means that repetitive brief ischemia has an influence on the whole body. Existent cells appear to have greater activity and cytokines accelerate vascular regeneration and tissue restored. Based on this, we hypothesized that repetitive brief ischemia can boost secretory ability of osteoblasts to promote bone healing. We built the tibia fracture models in rat by heavy percussion device in vivo experiment; then, affected limb received repetitive brief ischemia.

We found that repetitive brief ischemia can accelerate bone healing and the cell activity of osteoblast was accompanied by boosted BMP, ALP, and TGF-*β*1 using immune-histochemical staining in fracture area.

We are glad to find the benefit from repetitive brief ischemia on affected limb ischemia, but it is difficult to apply this method clinically. No matter how beneficial they will get, patients cannot tolerate the pain about ischemia operation on affected limb after surgery.

Fortunately, ischemia is not a simple physiological process in regions but also has an impact at every pore because we discovered the serum cytokines increased at the same time in models. Probably, repetitive brief ischemia on the healthy side limb can promote bone healing. This study was to verify this hypothesis.

We explored repetitive brief ischemia on bone healing by three parts, imaging, cytokines, and histology. Finally, we find there is no significant difference in the effects of promoting bone healing between repeated brief ischemia on the health side limb and on the affected side limb. Both of them can accelerate fracture healing. However, we think the mechanism is not the same. Repeated ischemia at the healthy side exerts promoting effect to the whole body, while repeated transient ischemia on the affected side also includes local factors.

Repetitive brief ischemia on the healthy side caused hemodynamic changes. The blood circulation volume increased in the rest of body when the health limbs were subjected to transient ischemia, especially in the fracture position. These changes may stimulate the reconstruction of local injury microcirculation. Besides, repetitive brief ischemia has impacts on the level of serum cytokines. Those cytokines can activate and mobilize the cells like mesenchymal stem cells and more cells are clustered in the fracture zone. Repetitive brief ischemia on the affected side made the hypoxia tolerance of many cells enhanced, which would promote the expression of a variety of genes that influenced tissue repair. The energy of cell storage in local tissues is increased, which is more conducive to tissue repair. Vascular endothelium is a key cell. Repetitive brief ischemia can accelerate the reconstruction of microcirculation and the activation of vascular endothelium is a factor as the VEGF increased.

The target of repetitive brief ischemia in promoting bone healing included periosteal osteogenesis and cartilaginous osteogenesis. It will accelerate the speed of osteogenesis of cartilage, which is reflected in the increase of the amount of callus ossification. Besides, the speed of periosteal osteogenesis is also enhanced due to the time of bone bridge passing fracture side shortened.

Maybe periosteum is also damaged when bone fracture happened. The osteogenesis of periosteum will also be strengthened as seen in the tissue pathological sections. This phenomenon may be associated with the cytokines secreted.

## 5. Conclusion

Repetitive brief ischemia affects the whole body and can accelerate bone healing. Besides, there was no significant difference in the effects of rehabilitation between repeated brief ischemia on the healthy side limb or the affected side.

## Figures and Tables

**Table 1:** Sequence of each group in observation results.

Observed indicator	Outcome
X-ray evaluation	
1 week	A24 = H24 > A48 = H48 = C24 = C48
2 weeks	A24 = H24 = A48 = H48 > C24 = C48
4 weeks	A24 = H24 = A48 = H48 = C24 = C48
Micro-CT evaluation	
BV	
2 weeks	A24 = H24 = A48 = H48 > C24 = C48
4 weeks	A24 = H24 = A48 = H48 > C24 = C48
TV	
2 weeks	A24 = H24 = A48 = H48 > C24 = C48
4 weeks	A24 = H24 = A48 = H48 = C24 = C48
BV/TV	
2 weeks	C24 = C48 > A24 = H24 = A48 = H48
4 weeks	A24 = H24 = A48 = H48 > C24 = C48
Cytokines results	
BMP-2	
1 week	A24 = H24 > A48 = H48 = C24 = C48
2 weeks	A24 = H24 = A48 = H48 > C24 = C48
4 weeks	A24 = H24 = A48 = H48 = C24 = C48
VEGF	
1 week	A24 = H24 > A48 = H48 = C24 = C48
2 weeks	A24 = H24 = A48 = H48 > C24 = C48
4 weeks	A24 = H24 = A48 = H48 = C24 = C48
IL-6	
1 week	A24 > A48 > H48 = C24 = C48 > H24
2 weeks	A24 > A48 > C24 =C48 > H48 > H24
4 weeks	A24 = H24 = A48 = H48 = C24 = C48
Diethanolamine enzyme activity unit of ALP	
1 week	A24 = H24 > A48 = H48 = C24 = C48
2 weeks	A24 = H24 = A48 = H48 > C24 = C48
4 weeks	A24 = H24 = A48 = H48 = C24 = C48
TGF-*β*1	
1 week	A24 = H24 > A48 = H48 = C24 = C48
2 weeks	A24 = H24 = A48 = H48 > C24 = C48
4 weeks	A24 = H24 = A48 = H48 = C24 = C48
IL-10	
1 week	H24 > H48 = A48 = C24 = C48 >A24
2 weeks	H24 > H48 > C24 = C48 > A48 >A24
4 weeks	A24 = H24 = A48 = H48 = C24 = C48

## Data Availability

The data used to support the findings of this study are available from the corresponding author upon request.
